# No Additive Effects of Polyphenol Supplementation and Exercise Training on White Adiposity Determinants of High-Fat Diet-Induced Obese Insulin-Resistant Rats

**DOI:** 10.1155/2018/7406946

**Published:** 2018-04-15

**Authors:** Karen Lambert, Marie Hokayem, Claire Thomas, Odile Fabre, Cécile Cassan, Annick Bourret, Florence Bernex, Jessica Lees, Marie Demion, Pascal Seyer, Gérald Hugon, Jacques Mercier, Antoine Avignon, Catherine Bisbal

**Affiliations:** ^1^PhyMedExp, CNRS UMR 9214, INSERM U1046, University of Montpellier, Montpellier, France; ^2^Départment STAPS, University of Evry, University of Paris Saclay, Evry, France; ^3^IRCM, Inserm, University of Montpellier, ICM, Montpellier, France; ^4^RHEM, BioCampus Montpellier, CNRS, INSERM, University of Montpellier, Montpellier, France; ^5^Centre Hospitalier Universitaire (CHU), Montpellier, France

## Abstract

One of the major insulin resistance instigators is excessive adiposity and visceral fat depots. Individually, exercise training and polyphenol intake are known to exert health benefits as improving insulin sensitivity. However, their combined curative effects on established obesity and insulin resistance need further investigation particularly on white adipose tissue alterations. Therefore, we compared the effects on different white adipose tissue depot alterations of a combination of exercise and grape polyphenol supplementation in obese insulin-resistant rats fed a high-fat diet to the effects of a high-fat diet alone or a nutritional supplementation of grape polyphenols (50 mg/kg/day) or exercise training (1 hr/day to 5 days/wk consisting of treadmill running at 32 m/min for a 10% slope), for a total duration of 8 weeks. Separately, polyphenol supplementation and exercise decreased the quantity of all adipose tissue depots and mesenteric inflammation. Exercise reduced adipocytes' size in all fat stores. Interestingly, combining exercise to polyphenol intake presents no more cumulative benefit on adipose tissue alterations than exercise alone. Insulin sensitivity was improved at systemic, epididymal, and inguinal adipose tissues levels in trained rats thus indicating that despite their effects on adipocyte morphological/metabolic changes, polyphenols at nutritional doses remain less effective than exercise in fighting insulin resistance.

## 1. Introduction

An obesogenic lifestyle of an excessive caloric intake versus a low energy expenditure via physical inactivity favors obesity by increasing fat mass. This detrimental state of obesity/extreme fatness is at the center of the pathophysiology of type 2 diabetes (T2D) by causing insulin resistance (IR) to appear several decades before T2D. T2D prevalence is increased with aging (prevalence of 12–25% among older adults (>65 years)) and is characterized by IR, metabolic alterations, and chronic inflammation [1]. The initiating mechanisms of IR are partially known with major inducers being oxidative stress and chronic inflammation [2–5]. Increased levels of reactive oxygen species and inflammatory cytokines have been found to negatively impact insulin signaling [1]. Interestingly, adipose tissue contributes to IR via adipocyte morphology dysfunction, adipocytokine secretion, or metabolic alterations. It has been shown that IR is linked to specific adipose tissue locations in the body [6]. In fact, the increase of metabolically active visceral adiposity is associated with IR development [7] while subcutaneous adiposity is associated with a lower risk of T2D development [8]. Consistent with the notion that subcutaneous fat is metabolically beneficial, its transplantation into the visceral cavity has been shown to improve insulin sensibility [9]. Exercise training, a potent insulin sensitizer, is associated with a greater loss of visceral versus subcutaneous fat [10, 11]. Only recently, investigations aiming at understanding the effects of exercise training on nonmuscle tissues and more specifically adipose tissue depots have been designed [10–13]. Exercise training has been shown to reduce adiposity, adipocyte size [10], oxidative stress [14], and inflammation levels [15] while inducing an increase in mitochondrial enzyme activities in white adipose tissue [16]. Thus, the positive health effects of exercise training in preventing metabolic chronic diseases could be due to its metabolic regulation of fat stores and not only to its undeniable impact on skeletal muscle metabolism.

As mentioned above, the health-promoting effects of physical activity are well documented, leading the scientific community to discover and even create “exercise-mimetic” molecules at the center of which are the phytochemical mega family of polyphenols. Polyphenols due to their antioxidant/anti-inflammatory properties [17] have been used to fight against IR with contradictory effects. Some studies report beneficial effects [18–22] while others do not [23–26]. In sedentary rodents, resveratrol, a polyphenol found in colored berries, induces metabolic adaptations normally seen in trained animals [19]. However, due to its very low concentration in fruits and vegetables, this polyphenol is not a good representative of dietary polyphenol intake [27]. Moreover, the fact that polyphenol-rich diets positively improve glucose homeostasis [28] and obesity parameters in an elderly population [29] highlights how polyphenols' health benefits cannot be attributed to a single compound but rather to a complex synergistic molecular effect. Accordingly to this data, we previously demonstrated the metabolic advantages of a mixture of grape polyphenol supplementation in nutritional amounts against fructose-induced IR and oxidative stress in healthy volunteers at high metabolic risk [30]. Interestingly, the combination of training and polyphenol supplementation on adipose tissue characteristics has been poorly investigated in obese IR rats [31]. The aim of our study was therefore to better understand the potential curative effects of a nutritional supplementation of grape polyphenols alone and in combination with exercise on the different adipose tissue depot characteristics in high-fat diet obese and IR rats in comparison with exercise training and grape polyphenol supplementation alone.

## 2. Materials and Methods

### 2.1. Ethical Approval

All animal experimentation procedures were in accordance with the Directive 2010/63/EU that was adopted on September 22, 2010, for the protection of animals used for scientific purposes (agreement number: A34–172-38) and were approved by the local research ethics committee (protocol number: CEEA-LR-1062).

### 2.2. Animals

After acclimation to the facility, a total of forty 6-week- (wk-) old male Sprague-Dawley rats (Janvier Laboratories) were fed a high-fat ad libitum diet (HFD, D12330 Research Diets, containing 25.5% carbohydrate, 58% fat, and 16.4% protein) for the 12-week duration of the experimental protocol. The rats were single or double housed in a temperature-controlled room and maintained with food and drink ad libitum in a 12 : 12 h light-dark cycle, lights on at 8 : 00 postmeridian. The training and *in vivo* tests were performed during the rats' dark cycle exposure. Rats' body mass was monitored every two days throughout the experimental period.

### 2.3. Experimental Design

**Figure 1:**
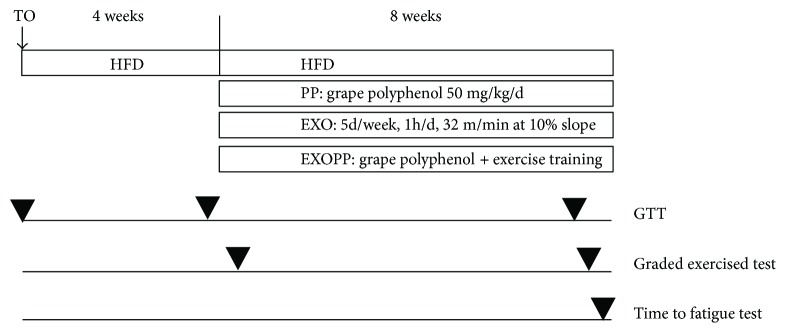
Experimental design. After 4 weeks of HFD, 40 male Sprague-Dawley rats were divided into 4 groups (*n* = 10/group) while simultaneously continuing for an 8-week period the HFD alone (HF), HFD, and supplementation with grape polyphenol extract (PP) at 50 mg/kg/d per 50 ml of drinking water (PP), HFD, and exercise training 1 hr per day/5 days/wk consisting of running on a treadmill set at 32 m/min for a 10% slope (EXO), HFD, and PP supplementation combined with exercise training (EXOPP). Glucose tolerance tests (GTT) were performed at T0, 4, and 11 weeks. A graded exercise test was completed between the 4th and the 5th week and between the 11th and the 12th week where a time-to-fatigue test was also done.

After 4 wks of HFD, previously shown to induce skeletal muscle IR [32], the animals were divided into 4 groups (*n* = 10/group) while simultaneously continuing the HFD for an additional 8-week period (Figure 1). Group specificities were the following: HFD alone (HF), HFD, and supplementation with grape polyphenol extract (PP) at 50 mg/kg/d per 50 ml of drinking water (PP), HFD, and exercise training 1 hr/day to 5 days/wk consisting of running on a treadmill set at 32 m/min for a 10% slope (EXO), HFD, and PP supplementation combined with exercise training (EXOPP). The grape polyphenol extract supplemented in this study (64.4% total polyphenol with 17.9% procyanidins, 4.3% anthocyanins, and 533 ppm resveratrol) had been previously successfully used in humans and in mice [30, 33]. The polyphenols' mixture intake was 33.0 ± 2.1 ml/d for PP and 31.9 ± 1.7 ml/d for EXOPP groups compared to a water intake of 26.5 ± 1.3 ml/d for HF and EXO groups, indicating that the polyphenol mixture was well accepted by the animals.

Glucose tolerance tests were performed on animals at the start of the protocol and then after 4 and 7 wks of HFD (Figure 1). During the first 4 wks, all animals were handled daily and familiarized 5 days a week/5–10 min/day with the treadmill set at 10 m/min at 10% slope. This exercise duration is insufficient to significantly increase aerobic performance [34, 35]. Graded exercise tests were performed after 4wks and then at 11 wks (Figure 1). The rats selected for the exercise groups (EXO and EXOPP) reached the required training intensity in 1 wk. Animals were euthanized 48 hrs after the last training session to avoid the acute effect of the last exercise training session. Euthanasia was done by exsanguination after isoflurane anesthesia on 12 hr-fasted animals. Immediately after death, epididymal, perirenal, inguinal, anterior subcutaneous, and mesenteric adipose tissues were rapidly excised as previously detailed [36], weighed, and directly frozen in liquid nitrogen. Total fat mass (g) was estimated by summing the weights of the different fat stores excised. Adiposity (%) was calculated as the sum of the different adipose tissue depots (g) devised by rat's body weight and multiplied by 100. Soleus, tibialis anterior, and extensor digitorum longus muscles were also rapidly surgically removed and weighted to determine lean mass.

### 2.4. Exercise Tests

#### 2.4.1. Graded Exercise Test (GET)

This test consisted of a progressive exercise test in which each rat initially ran at a speed of 10 m/min, up a 5% slope, for 3 min. Thereafter, the speed was increased by 3 m/min every 2 min until the rat was unable/unwilling to keep pace with the treadmill belt despite encouragement to do so by application of manual solicitation at the hind legs.

### 2.5. Blood Analysis

#### 2.5.1. Glucose Tolerance Test (GTT)

After a 12 hr fast, rats were injected intraperitoneally with a 30% glucose solution (2 g/kg body weight), as previously described [37]. Glucose levels were determined using QuantiChrom™ Glucose Assay Kit (BioAssay Systems) on blood collected from the tail, at baseline and following glucose injection. Insulin levels were determined at baseline using Ultrasensitive Insulin ELISA (Mercodia). Homeostasis model assessment of IR (HOMA-IR) index was calculated using the formula: HOMA-IR = [fasting insulin (*μ*IU/ml) × fasting glucose (mmol/ml)]/22.5. Kg represented the exponential decrease in glycaemia during glucose tolerance test and thus glucose disposal [38].

#### 2.5.2. Determination of Free Fatty Acid, Total Cholesterol, Low- and Very Low-Density Lipoproteins, High-Density Lipoproteins, and Leptin Plasma Levels

Plasma samples from 12 hr-fasted rats were collected under anesthesia from the carotid artery and centrifuged for 10 min at 2000 ×g. Free fatty acid (FFA), total cholesterol, low- and very low-density lipoproteins (LDL/VLDL), and high-density lipoproteins (HDL) levels were determined using EnzyChrom™ Free Fatty Acid Assay Kit (EFFA, BioAssay System) and EnzyChrom HDL and LDL/VLDL Assay Kit (EHDL, BioAssay System). Leptin concentration was quantified by ELISA (BioVendor).

### 2.6. Adipose Tissue Analysis

Adipose tissue insulin response was assessed by measuring P-Akt/Akt protein *ratio* in epididymal, inguinal, peritoneal, and mesenteric adipose portions incubated or not with insulin, as previously described [39, 40]. Two 50–100 mg pieces of adipose tissue slices were washed in phosphate buffer saline (PBS). Then, one entire explant was incubated at 37°C for 15 min with PBS only or supplemented with 1 *μ*M human insulin (Umuline RAPIDE) [30] before being snap frozen in liquid nitrogen. The adipose tissue portions were disrupted and homogenized in a hypotonic lysis buffer [41] with protease and phosphatase inhibitors (Sigma-Aldrich). Protein concentration was measured in the extracts using the Pierce BCA Protein Assay Kit (ThermoFisher Scientific). Protein expression was assessed on protein extracts by Western blot, using antibodies diluted in Odyssey Blocking Buffer (LI-COR Biosciences, Bad Homburg, Germany). Insulin response was determined using anti-Phospho-Akt (P-Akt) (Ser473) and anti-Akt antibodies at a 1 : 1000 dilution (both from Cell Signaling Technology Inc). Similarly, phospho-AMPK (P-AMPK, Thr172), AMPK, I*κ*B*α*, and SOCS3 proteins expression were analyzed in adipose tissues using anti P-AMPK, AMPK at a 1 : 500 and 1 : 1000 dilution, anti-I*κ*B*α* primary antibody at a 1 : 500 dilution, anti-SOCS3 primary antibody at a 1 : 1000 dilution, and *α*/*β*-tubulin antibody at a 1 : 1000 dilution (all five antibodies used were from Cell Signaling Technology Inc.). Following primary antibody incubation, nitrocellulose membranes were incubated with secondary antibodies that were directed against the species of the primary antibody while also being conjugated to IRDye800 or IrDye700, at a 1 : 30,000 dilution (LI-COR Biosciences). Specific bands were visualized with a LI-COR Odyssey CLx Imaging System (LI-COR Biosciences). Protein expression levels were quantified using ImageJ software. Finally, levels of protein expression in each sample were corrected using the respective quantified levels of the corresponding loading control (Akt for P-Akt, AMPK for P-AMPK, or *α*/*β*-tubulin for I*κ*B*α* and SOCS3) analyzed on the same membrane.

### 2.7. Adipocyte Size

Relative adipocyte size was estimated at 20-fold magnified hematoxylin and eosin-stained sections by counting the number of adipocytes in 5 random fields of each sample with two sections by sample. Relative cell size was expressed as adipocyte count per section. An increase in adipocyte count per section is related to a decrease in adipocyte size.

### 2.8. Citrate Synthase (CS) Activity

Enzymatic activity of citrate synthase was determined by spectrometry, as previously described on soleus homogenates [42].

### 2.9. Statistical Analysis

Data are expressed as means ± standard error of the mean (SEM). Comparisons between treatment groups were performed using two-way analysis of variance (ANOVA) with a Bonferroni post hoc test of pairwise comparisons between groups or using three-way analysis of variance (ANOVA) with a Bonferroni post hoc test of pairwise comparisons between groups or Student's unpaired, two-tailed *t*-test, when required. A probability value of <0.05 was considered significant.

## 3. Results

### 3.1. Animal Characteristics

**Figure 2:**
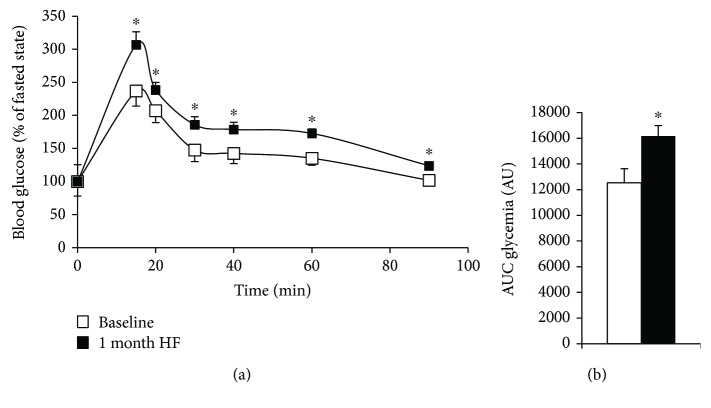
Glucose tolerance test scores. (a) Glycaemia expressed in percentage of basal glycaemia and (b) area under the curve (AUC) during glucose tolerance test of 16 male Sprague-Dawley rats before (baseline, white squares) and after 4 weeks of an ad libitum high-fat diet (black squares). Data are expressed as mean ± SEM. Significant difference: ^∗^*p* < 0.05 versus baseline.

After one month of the high-fat diet (HF), rats presented an increase in both body weight 250.6 g ± 1.7 to 422.2 g ± 4.8 (*p* < 0.001) and glycaemia during glucose tolerance test measurements compared to baseline (*p* < 0.05 for all points) (Figures 2(a) and 2(b)) thus demonstrating an impairment in glucose disposal associated with obesity. During the eight following weeks, the animals were subjected to high-fat diet alone (HF) or HF with a supplementation of grape polyphenol extract at nutritional doses (PP) or to exercise training (EXO) or to the combination of exercise and polyphenol supplementation (EXOPP). As expected, we observed an increase in treadmill speed during the graded exercise test (GET) and an increase in muscle citrate synthase activity in trained rats compared to sedentary rats (Table 1). Free fatty acid (FFA) were decreased only in trained rats (EXO and EXOPP versus HF and PP, *p* < 0.05), and high-density lipoproteins (HDL) were increased after polyphenol supplementation (PP and EXOPP versus HF and EXO, *p* < 0.05) (Table 1). Total cholesterol, low-density lipoproteins (LDL), and very low-density lipoproteins (VLDL) levels remained unaltered in all experimental groups tested (Table 1).

### 3.2. Metabolic Characterization

**Figure 3:**
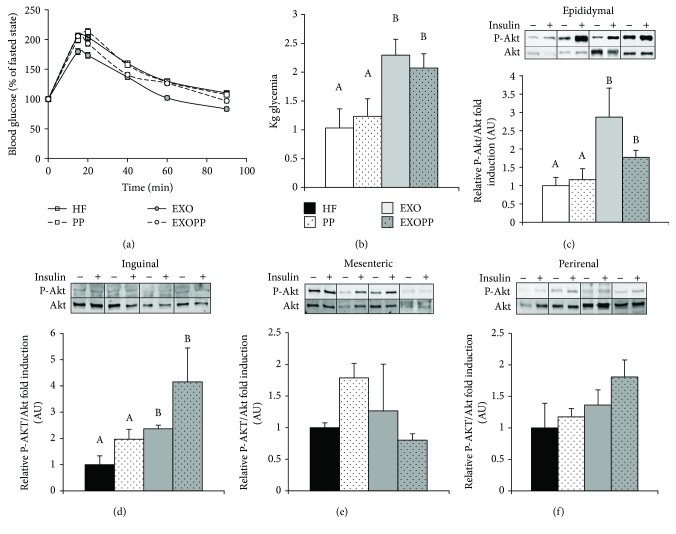
Measure of whole-body glucose homeostasis and adipose tissue insulin sensitivity. (a) Glycaemia expressed in percentage of fasted blood glucose and (b) glucose disposal (Kg) calculated from GTT. Data are expressed as mean ± SEM with *n* = 8–10 rats per group. P-Akt and Akt expression levels were measured by immunoblotting after insulin stimulation in epididymal (c), inguinal (d), mesenteric (e), and perirenal (f) fat stores. A representative blot is shown for each adipose tissue depot. Quantified values are presented as P-Akt/Akt fold induction relative to HF group's values, which were set at 1. Data are expressed as mean ± SEM with *n* = 5 rats per group. Bars not sharing a common letter are significantly different at *p* < 0.05.

**Figure 4:**
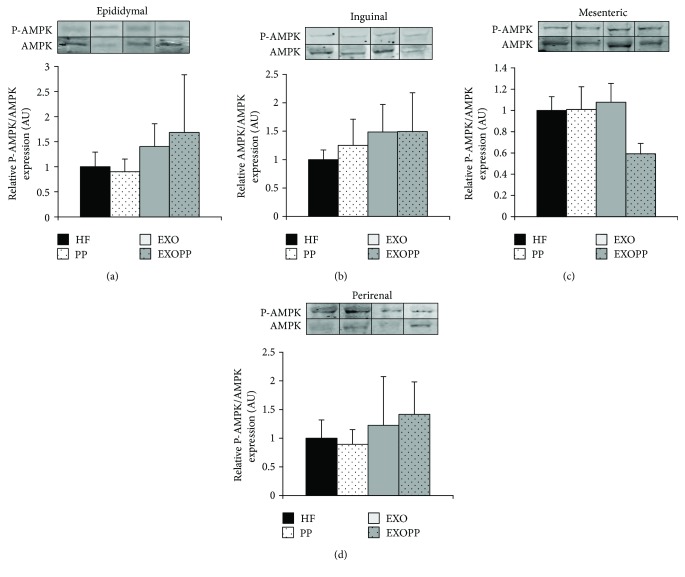
Effect of exercise training and polyphenol supplementation on P-AMPK/AMPK levels in epididymal, inguinal, mesenteric, and perirenal fat stores. P-AMPK and AMPK expression levels were measured by immunoblotting in epididymal (a), inguinal (b), mesenteric (c), and perirenal (d) fat stores. A representative blot is shown for each adipose tissue depot. Values are measured as P-AMPK/AMPK ratios relative to HF group's values, which were set at 1. Data are expressed as mean ± SEM with *n* = 4 rats per group.

Whole-body glucose tolerance was determined with a glucose tolerance test (GTT) (Figure 3(a)). Glucose disposal during GTT was increased in trained rats (EXO and EXOPP versus HF and PP) as attested by increased of Kg glycaemia constant (Figure 3(b), *p* < 0.05), indicating that whole-body glucose tolerance was improved by exercise training (EXO and EXOPP versus HF and PP). Moreover, EXOPP rats presented the lowest HOMA-IR compared to HF, PP, and EXO rats, demonstrating a higher insulin sensitivity (Table 1). To further our understanding, we investigated the insulin response of each adipose tissue depots obtained (Figures 3(c), 3(d), 3(e), and 3(f)) by measuring P-Akt/Akt *ratio* before and after incubation with insulin. P-Akt/Akt ratio was increased only in epididymal and inguinal fat stores of trained rats (EXO and EXOPP versus HF and PP). When analyzed together, all adipose tissues collected presented significant increases in P-Akt/Akt ratios following exercise training. Alone, polyphenol supplementation did not increase P-Akt/Akt ratio. We also investigated P-AMPK/AMPK activation in the epididymal, inguinal, mesenteric, and perirenal adipose tissue depots (Figures 4(a), 4(b), 4(c), and 4(d)) in order to evaluate GLUT4 translocation [43]. We found no notable change in P-AMPK/AMPK ratio in any of the experimental conditions (exercise and/or polyphenol supplementation) or any of the adipose tissue depots.

### 3.3. Adipose Tissue Depot Characteristic Modification

**Figure 5:**
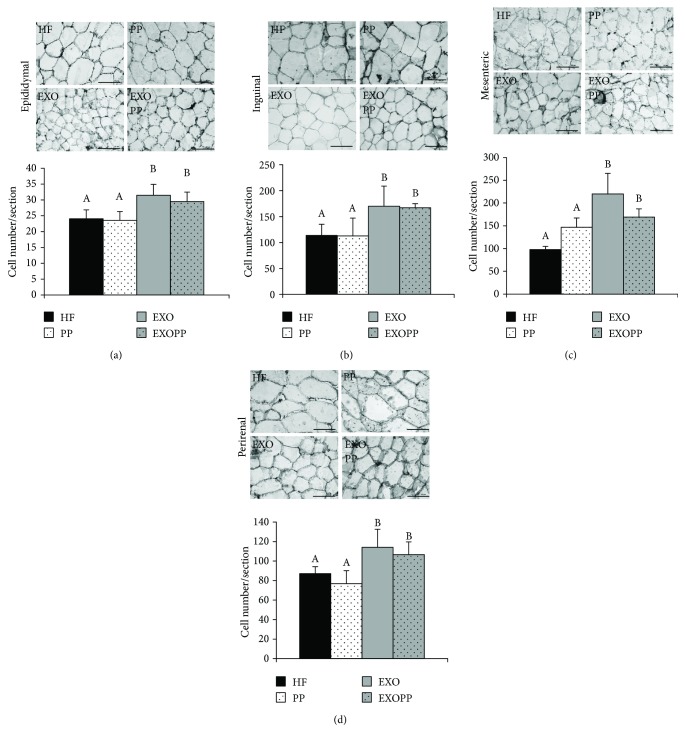
Effect of exercise training and polyphenol supplementation on adipose tissue. Representative sections of epididymal (a), inguinal (b), mesenteric (c), and perirenal (d) adipose tissues stained with hematoxylin and eosin are shown. Relative cell size was expressed as adipocyte count per section. An increase in adipocyte count per section is related to a decrease in adipocyte size. Data are expressed as mean ± SEM with *n* = 8–10 rats per group. Bars not sharing a common letter are significantly different at *p* < 0.05.

After eight weeks of high-fat diet associated with exercise training (EXO) alone or combined with polyphenol supplementation (EXOPP), trained rats had a decrease in body weight compared to sedentary rats (HF and PP), without any beneficial effect of polyphenol supplementation on this parameter (Table 1). This decrease is mainly due to a reduced fat mass (Table 2) that is significantly correlated to body weight (*r* = 0.726, *p* < 0.0001). Total fat mass decrease was observed in exercise-trained groups (EXO and EXOPP, *p* < 0.001) (Table 2) with a tendency to decrease also observed in the PP group (*p* = 0.059, Table 2). Although not significantly different from EXO rats, EXOPP rats exhibited the smallest fat mass and adiposity values compared to all other animal groups (Table 2). Exercise training decreased the quantity of all fat pads: inguinal, epididymal, perirenal, mesenteric, and anterior subcutaneous (Table 2). In addition, as shown in Figures 5(a), 5(b), 5(c), and 5(d), exercise training significantly increased adipocyte number per section (*p* < 0.05) in all adipose tissue depots which indicates an overall decrease in adipocyte size. When combined together, the three-way ANOVA indicated that polyphenol supplementation lowered the mass of all types of white adipose tissue studied (Table 2, *p* = 0.006). An interaction was also found between all adipose tissue depots and exercise training (*p* = 0.003). Nevertheless, the amplitude of the decrease was not the same for all fat stores with inguinal and mesenteric adipose tissues presenting the lowest decrease due to exercise training, as opposed to anterior subcutaneous, perirenal, and epididymal adipose tissues. We investigated visceral fat pad (total sum of epididymal, perirenal, and mesenteric adipose tissues) weight as it has previously been shown that during high-fat feeding, exercise training preferentially affects this type of fat store [10]. Here, due to exercise training, we observed a decrease in visceral adipose tissue (*p* < 0.001) and subcutaneous adipose tissue stores (*p* < 0.033) (Table 2). Combination of polyphenol supplementation with exercise training did not further amplify this reduction compared to exercise alone. Interestingly, we found an important decrease in the ratio of fat mass on lean (muscle) mass with training. The effect of polyphenol supplementation was close to significance (*p* = 0.056), which implies that polyphenol supplementation, as exercise, may induce a decrease in fat mass associated with an increase in muscle mass.

### 3.4. Leptin Levels, I*κ*B, and SOCS3 Expression According to White Adipose Tissue Fat Depot Type

**Figure 6:**
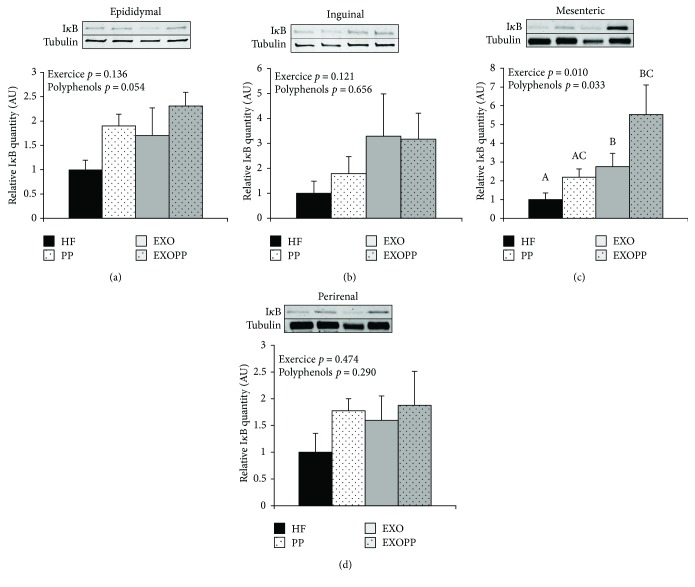
I*κ*B levels in fat stores. I*κ*B protein expression levels were measured by immunoblotting in epididymal (a), inguinal (b), mesenteric (c), and perirenal (d) fat stores. Quantification values are presented as I*κ*B protein levels normalized to tubulin protein level, used as protein loading control, and are expressed relative to HF group's values, which were set at 1. Data are expressed as mean ± SEM with *n* = 5 − 6 rats per group. Bars not sharing a common letter are significantly different at *p* < 0.05.

**Figure 7:**
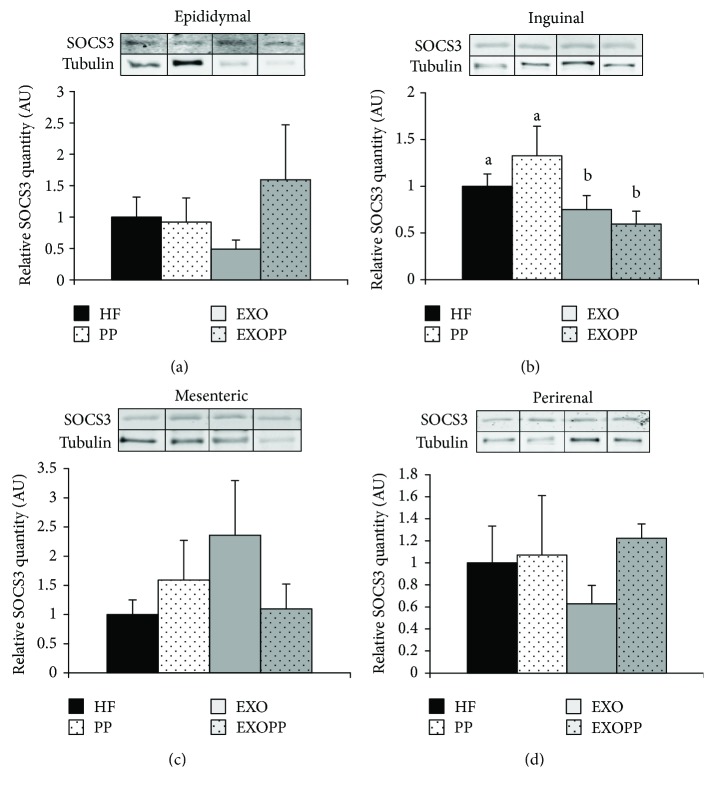
SOCS3 levels in fat stores. SOCS3 protein expression levels were measured by immunoblotting in epididymal (a), inguinal (b), mesenteric (c), and perirenal (d) fat stores. Quantification values are presented as SOCS3 protein levels normalized to tubulin protein levels, used as protein loading control, and are expressed relative to HF group's values, which were set at 1. Data are expressed as mean ± SEM with *n* = 4 − 5 rats per group. Bars not sharing a common letter are significantly different at *p* < 0.05.

Leptin is a major adipokine related to the amount of energy stored as fat in white adipose tissue and involved in the pathogenesis of chronic inflammation [44]. Interestingly, we observed a decrease in leptin levels after training (Table 2); however, leptin/adiposity ratio was constant between groups (Table 2), indicating that the decrease in leptin levels must be related to adiposity decline. Confirming this result, we found strong correlations between leptin levels and both subcutaneous (*R* = 0.668, *p* < 0.00001) and visceral (*R* = 0.628, *p* < 0.0001) fat pad mass. Seeing that leptin is at the interface between metabolism and inflammation [44], we investigated I*κ*B protein levels in the different adipose tissue depots as I*κ*B is able to sequester the transcription factor NF*κ*B in the cytosol and thus counteracting its proinflammatory action [45] (Figures 6(a)–6(d)). Individually, only mesenteric fat stores presented an increase in I*κ*B protein levels due to both exercise (*p* = 0.010) and polyphenol supplementation (*p* = 0.033) (Figure 6(c)). However, when analyzed together, the three-way ANOVA showed a significant effect of both exercise (*p* < 0.001) and polyphenol supplementation (*p* = 0.005). Thus, both interventions were able to improve adipose tissue inflammation due to high-fat feeding. Again, there was no additive effect of exercise and polyphenol on this parameter. Since suppressor of cytokine signaling 3 (SOCS3) is a protein involved in a negative feedback loop of cytokine activity [46] but also in insulin signaling regulation [47], we explored its expression in the different adipose tissue depots (Figures 7(a)–7(d)) and found that SOCS3 protein content only significantly decreased in inguinal fat depots (*p* = 0.040) (Figure 7(b)) of trained rats. Polyphenol supplementation did not modify SOCS3 protein content in any adipose tissue depot studied.

## 4. Discussion

In this study, we investigated, in high-fat IR obese rats, the potential curative effects of exercise training combined or not with a daily nutritional supplementation of a grape polyphenol mixture on different adipose tissue depots. The main result of our study is that compared to exercise alone, combining exercise training to a supplementation of polyphenols has no cumulative beneficial effect on adipose tissue alterations of IR obese rats whatever their location.

In our model of obese IR rats, exercise training decreases the total amount of white adipose tissue with a more significant decrease in visceral (*p* < 0.001) than subcutaneous (*p* < 0.05) depots. Interestingly, polyphenol supplementation also induces a decrease in the total amount of adipose tissue depots. However, even though there is an improvement in insulin sensitivity at both systemic and epididymal/inguinal adipose tissue levels with exercise training, such improvement is not observed with polyphenol supplementation alone. Thus, the reduction in adipose tissue mass observed with our nutritional polyphenol supplementation is not associated with an improvement of insulin sensitivity at both systemic and tissular levels as observed in previous study reporting a decrease in body weight without improvement of insulin sensitivity [48]. However, in our study, such surprising result could be due to an insufficient decrease in fat pads with polyphenol supplementation (−22% with PP versus −40% with EXO). Another hypothesis is that the beneficial effect of exercise training on adipose tissue is not only related to its lowering effect on adipose tissue mass and adipocyte size but may involve other mechanisms. Interestingly, we extend in obese insulin-resistant rat the previous data from Stanford et al. [13] showing that exercise training was able to remodel inguinal adipose tissue in normal weight rats. Moreover, we did not find significant variation in AMPK activation in any adipose tissue location; even if there is a tendency of P-AMPK/AMPK ratio to increase after training.

Adipose tissue was previously thought to be a mere storage depot for lipids and energy; several studies have now shown that adipocytes secrete biologically active molecules, that is, adipokines as leptin with complex autocrine, paracrine, and endocrine functions [49]. A dysregulation in such adipokine levels and leptin resistance has been implicated in the physiopathology of IR and T2D [50]. However, we have recently shown that such dysregulation was not found in women with grade I obesity since IR and insulin sensitive women presented the same adipokine levels [39]. As previously shown in several human studies [51–53], our results point out that leptin is not involved in the beneficial effect of exercise as there is no difference in leptin levels between groups when adjusted to adipose tissue mass. However, the decreased adipocytes' size observed with exercise training could participate in the improvement of insulin sensitivity. In accordance with this data, we did not observe an improvement in insulin sensitivity with polyphenol supplementation or a decrease in adipocyte size. In fact, large adipocytes have been associated with ROS production leading to IR and inflammatory reactions [54, 55]. Exercise training and polyphenol supplementation improve only mesenteric adipose tissue inflammation whereas there is no improvement in insulin sensitivity in this fat pad depot. The three-way ANOVA demonstrates that the other adipose tissue depots also present metabolic improvement with exercise only when analyzed together. SOCS3 diminishes insulin-stimulated IRS1 phosphorylation leading to a decrease in insulin response [56–58]. Interestingly, we observed only in inguinal fat depots a decrease in SOCS3 protein content in trained rat which is associated with an increase in insulin sensitivity in this fat depot after training. Thus, mesenteric fat stores are more sensitive to our polyphenol supplementation while inguinal and epididymal adipose tissue depots are more sensitive to training, demonstrating a depot-specific response as previously found by others [15, 59].

Our polyphenol supplementation presents some similar beneficial effects of exercise training as a reduction of adipose tissue depots and of mesenteric inflammation, which has been both described as positive regulators of insulin sensitivity. More interestingly, exercise training has been shown in our study to improve systemic and adipose tissue insulin sensitivity while the combination of exercise and polyphenol supplementation (EXOPP) results in an improvement in systemic insulin sensitivity attested by a significant decrease in HOMA-IR without further alterating any adipose tissue markers tested in this study.

Taken together, our data seem to indicate that the beneficial effects of exercise training are not only due to alteration in adipose tissue. Recent data in mice [60] and obese patients [61] also confirm that adipose tissue alterations in the development of insulin resistance and T2D play a less important role than once thought. We previously found in obese women that skeletal muscle seems to be the primary tissular target of insulin signaling defects in obesity development [39]. Exercise training and polyphenol supplementation individually or in combination have been found to increase insulin sensitivity at whole-body but also at skeletal muscle levels [30, 62, 63]. Thus, the improvement of systemic insulin sensitivity that we observed after training could also be due to its effect on skeletal muscle.

In conclusion, our study indicates that in high-fat diet-induced obese and IR rats, the combination of polyphenol supplementation with exercise training presents no more cumulative benefit on white adipose tissue alterations than exercise training or polyphenol supplementation alone.

## Figures and Tables

**Table 1:** Metabolic changes after polyphenol supplementation and exercise training.

	HF	PP	EXO	EXOPP	Two-way ANOVA
Weight (g)	603 ± 19	570 ± 15	539 ± 15 ^∗^	542 ± 15 ^∗^	Exercise *p* < 0.01
Treadmill speed (m/min)	28 ± 1.0	28 ± 1.3	41.5 ± 1.5	42.25 ± 1.1	Exercise *p* < 0.01
Leptin/adiposity	0.26 ± 0.02	0.19 ± 0.03	0.23 ± 0.03	0.21 ± 0.03	NS
HOMA-IR	2.3 ± 0.1	2.2 ± 0.2^$^	2.1 ± 0.2^$^	1.7 ± 0.1^∗^	Exercise *p* < 0.01
FFA (*μ*M)	346.6 ± 77.1	218.9 ± 27.0	147.0 ± 31.6	174.7 ± 36.2	Exercise *p* < 0.05
HDL (mg/dl)	28.8 ± 1.8	31.1 ± 2.3	30.5 ± 2.3	36.7 ± 1.6^∗^	Exercise *p* = 0.074 Supplementation *p* < 0.05
Cholesterol (mg/dl)	62.8 ± 3.6	63.1 ± 3.3	57.7 ± 7.2	69.7 ± 4.3	*p* > 0.05
LDL/VLDL (mg/dl)	28.7 ± 2.7	24.5 ± 2.8	29.2 ± 3.0	26.1 ± 2.7	*p* > 0.05
Citrate synthase activity (*μ*mol/min/g tissue)	13.7 ± 0.7	16.9 ± 1.2	19.9 ± 1.9	16.5 ± 0.4	Exercise *p* < 0.05

^∗^ *p* < 0.05 versus HF; ^$^*p* < 0.05 versus EXOPP.

**Table 2:** Impact of polyphenol supplementation and exercise on fat stores.

	HF	PP	EXO	EXOPP	Two-way ANOVA	Three-way ANOVA
Fat mass (g)	63.5 ± 5.5	49.8 ± 4.9	38.0 ± 4.3	35.1 ± 3.2	Exercise *p* < 0.001 Suppl. effect *P* = 0.059	NA
Adiposity (%)	10.1 ± 0.7	8.7 ± 0.7^$^	6.4 ± 0.5 ^∗^	6.2 ± 0.5 ^∗^	Exercise *p* < 0.001	NA
Leptin (ng/ml)	2.5 ± 0.4	1.6 ± 0.2^∗^	1.4 ± 0.1^∗^	1.2 ± 0.1^∗^	Exercise and suppl. effect *p* < 0.05	NA
Inguinal fat (g)	9.2 ± 1.5	4.9 ± 1.17.507	3.9 ± 1.8	3.6 ± 1.7	Exercise *p* = 0.056	Suppl. effect *p* = 0.006 interaction fat stores and exercise *p* = 0.003
Epididymal fat (g)	15.3 ± 1.0	13.3 ± 1.2^$^	9.0 ± 1.2^∗^	8.4 ± 1.2^∗^	Exercise *p* < 0.001	
Perirenal fat (g)	20.7 ± 1.6	16.6 ± 1.8^$^	11.4 ± 1.9^∗^	10.3 ± 1.8^∗^	Exercise *p* < 0.001	
Mesenteric fat (g)	17.2 ± 1.1	14.2 ± 1.2	13.1 ± 1.3^∗^	12.4 ± 1.2^∗^	Exercise *p* < 0.05	
Anterior subcutaneous fat (g)	1.2 ± 0.1	0.9 ± 0.1^$^	0.6 ± 0.1^∗^	0.5 ± 0.1^∗^	Exercise *p* = 0.001	
Visceral fat (g)	53.2 ± 4.0	44.0 ± 4.4^$^	33.5 ± 3.7 ^∗^	31.1 ± 2.9 ^∗^	Exercise *p* < 0.001	NA
Subcutaneous fat (g)	10.3 ± 1.6	5.8 ± 1.7	4.5 ± 1.8 ^∗^*p* = 0.091	4.0 ± 1.7^∗^	Exercise *p* = 0.033	NA
Fat mass/lean mass^∗^100	4.5 ± 0.4	3.4 ± 0.3 ^∗$^	2.3 ± 0.2^∗^	2.2 ± 0.2^∗^	Exercise *p* < 0.001 Suppl. effect *p* = 0.056	NA

^∗^ *p* < 0.05 versus HF, ^$^*p* < 0.05 versus EXOPP. Suppl. effect: polyphenol supplementation effect. Exercise: exercise training effect. NA: not applicable
